# Supplementary material for: The causal relationship between the human gut microbiota and pyogenic arthritis: a Mendelian randomization study
Source: Front Cell Infect Microbiol. 2024 Nov 26;14:1452480. doi: 10.3389/fcimb.2024.1452480 (PMC11629706; doi:10.3389/fcimb.2024.1452480)
Supplement: Supplementary file 1 [file DataSheet1.zip › mendelian test/ebi-a-GCST90027836.csv_forest.pdf]

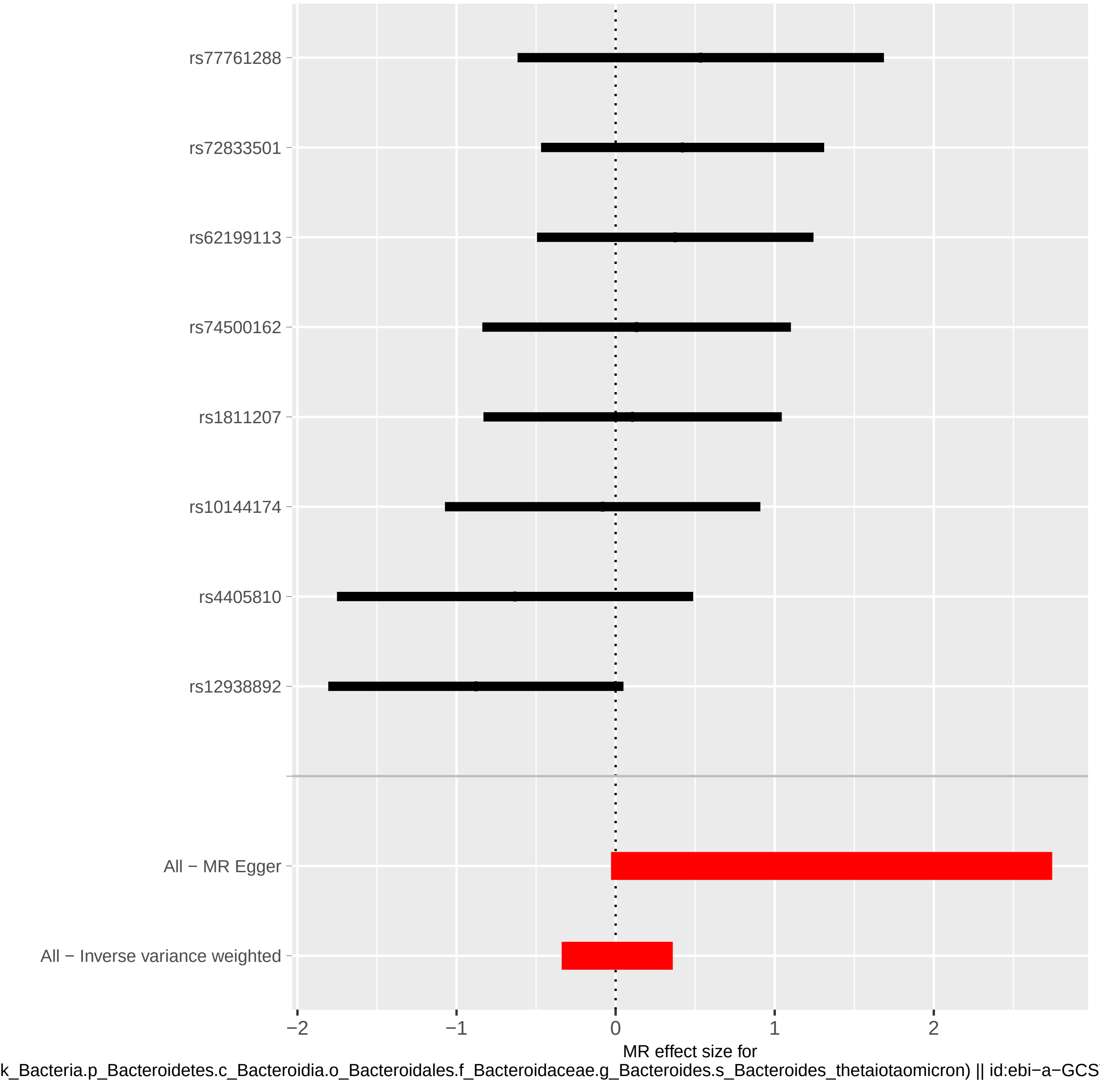
rs77761288
rs72833501
rs62199113
rs74500162
rs1811207
rs10144174
rs4405810
rs12938892
All − MR Egger
All − Inverse variance weighted
−2
−1
0
1
2
MR effect size for
k_Bacteria.p_Bacteroidetes.c_Bacteroidia.o_Bacteroidales.f_Bacteroidaceae.g_Bacteroides.s_Bacteroides_thetaiotaomicron) || id:ebi−a−GCS
